# Translation initiation complex eIF4F is a therapeutic target for dual mTOR kinase inhibitors in non-Hodgkin lymphoma

**DOI:** 10.18632/oncotarget.3378

**Published:** 2015-03-20

**Authors:** Christos Demosthenous, Jing Jing Han, Mary J. Stenson, Matthew J. Maurer, Linda E. Wellik, Brian Link, Kristen Hege, Ahmet Dogan, Eduardo Sotomayor, Thomas Witzig, Mamta Gupta

**Affiliations:** ^1^ Division of Hematology, Department of Internal Medicine, Mayo Clinic, Rochester, MN, USA; ^2^ Department of Internal Medicine, University of Iowa College of Medicine, IA, USA; ^3^ Celgene Corporation, San Francisco, CA, USA; ^4^ Department of Pathology, Memorial Sloan-Kettering Cancer Center, New York, NY, USA; ^5^ Department of Malignant Hematology, H. Lee Moffitt Cancer Center, Tampa, FL, USA

**Keywords:** translation initiation complex, eIF4E, lymphoma, dual mTOR inhibitors, CC214-1

## Abstract

Deregulated mRNA translation has been implicated in disease development and in part is controlled by a eukaryotic initiation complex eIF4F (composed of eIF4E, eIF4G and eIF4A). We demonstrate here that the cap bound fraction from lymphoma cells was enriched with eIF4G and eIF4E indicating that lymphoma cells exist in an activated translational state. Moreover, 77% (110/142) of diffuse large B cell lymphoma tumors expressed eIF4E and this was associated with an inferior event free survival. Over-expression of wild-type eIF4E (eIF4E^WT^) but not cap-mutant eIF4E (eIF4E^cap mutant^) increased the activation of the eIF4F complex. Treatment with the active-site dual mTOR inhibitor CC214-1 reduced the level of the eIF4F complex by decreasing the cap bound fraction of eIF4G and increasing the levels of 4E-BP1. CC214-1 inhibited both the cap dependent and global protein translation. CC214-1 inhibited c-Myc, and cyclin D3 translation by decreasing polysomal fractions from lymphoma cells. Inhibition of eIF4E with shRNA further decreased the CC214-1 induced inhibition of the eIF4F complex, c-Myc, cyclin D3 translation, and colony formation. These studies demonstrate that the eIF4F complex is deregulated in aggressive lymphoma and that dual mTOR therapy has therapeutic potential in these patients.

## INTRODUCTION

Non-Hodgkin lymphoma (NHL) is the 7th most common cause of cancer in the USA with diffuse large B-cell lymphoma (DLBCL) being the most common type in the US.[1] With standard RCHOP therapy approximately 60% of patients are cured; however, 40% of patients relapse and die of disease. New treatments are needed that target the specific signal pathways that are activated in lymphoma cells to enhance the initial response and prevent relapse. Recent reviews have described the potential importance of therapies that target protein translation.[2, 3] The translation of mRNA to protein is controlled by the eIF4F complex, a critical regulator of cap-dependent translation in eukaryotes. The eIF4F complex contains a translation initiation factor 4E (eIF4E), a scaffolding protein eIF4G, and the RNA helicase eIF4A. eIF4E is the cap-binding factor and its overexpression in solid tumor cells is associated with higher rates of cancer recurrence and cancer-related death.[4, 5] Various studies have demonstrated the overexpression of eIF4E in solid malignancies including esophageal cancer [6] and breast cancer;[7] however, there are currently no available FDA-approved anti-cancer agents that directly target eIF4E.

eIF4E is controlled by 4E-binding proteins (4E-BPs) that are one of the important downstream targets of the mTOR pathway.[8] Hyper-phosphorylated 4E-BPs bind weakly to eIF4E allowing eIF4E to bind with eIF4G and activate mRNA translation. It has been previously demonstrated that targeting 4E-BP1 phosphorylation using inhibitors of mTORC1 such as rapamycin analogues results in important but modest clinical responses.[9, 10] *In vitro* studies have attributed this partial and transient response to rapalogs to additional changes that occur, including high expression of mTORC2 and subsequent Akt and eIF4E phosphorylation.[11] To improve on the clinical results with single-agent mTORC1 inhibitors, combination therapies and dual mTOR inhibitors that target both mTORC1 and mTORC2 have been developed. These types of inhibitors compete with ATP in the catalytic site of mTOR, inhibiting the function of both mTORC1 and mTORC2 and blocking the feedback Akt and eIF4E activation.[12] We recently demonstrated that the cytotoxic and antiproliferative effects of dual mTOR inhibition were more effective than rapamycin at inhibiting malignant cell proliferation and inducing apoptosis.[13] The present study was designed to comprehensively evaluate the role of the eIF4F complex in aggressive lymphoma cell growth and learn if dual mTOR inhibitor could inhibit eIF4F complex mediated mRNA translation.

## RESULTS

### Integrity of the translation initiation complex eIF4F in lymphoma cells

**Figure 1:**
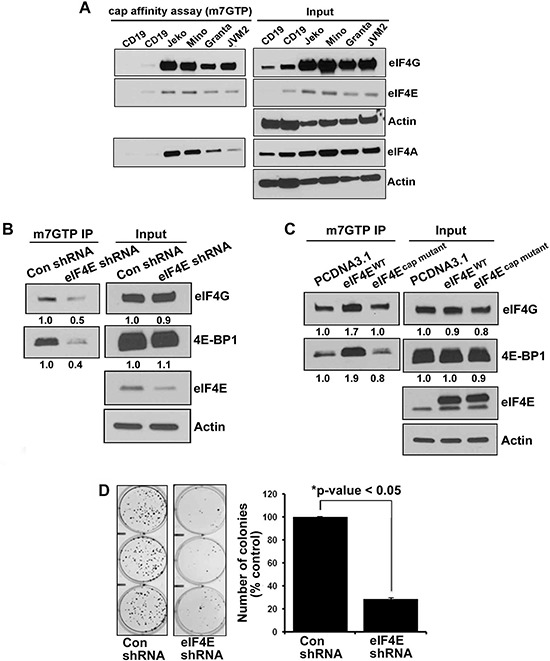
Integrity of eIF4F complex in normal B cells and lymphoma cells **(A)** *In vitro* cap affinity assay was performed in 4 MCL cell lines (as indicated in the figure) and CD19+ normal B cells and western blotting was performed using eIF4G, eIF4A and eIF4E antibodies. **(B)** Effect of eIF4E inhibition on eIF4F complex integrity was assessed in the lysates from stably transfected HEK293^eIF4E/KO^ and HEK293^control shRNA^ cells by the cap affinity assay. The experiment was performed in triplicate with similar results **(C)** *In vitro* cap affinity assay in HEK293 cells after transient overexpression of eIF4E^WT^ and eIF4E^cap mutant^. The experiment was performed in triplicate. **(D)** Colony-forming assay was performed in stably transfected HEK293^eIF4E/KO^ and HEK293^control shRNA^ cells. Bars represent mean ± SD from 3 replicates. The experiment was performed in triplicate.

We assessed the formation of the active eIF4F (m7GTP-eIF4E-eIF4G) translation initiation complex by a pull down assay using an agarose-immobilized m7GTP cap analog to capture eIF4E and its binding partners eIF4G and eIF4A in MCL cell line cells. The relative amount of captured eIF4G or eIF4A serves as an indicator of the integrity of the eIF4F translation complex. Cell lysates from Jeko, Mino, Granta, JVM2 and CD19+ normal B cells were incubated with m7GTP and analyzed by immunoblotting for the level of eIF4A and eIF4G. Our data demonstrate that the cap-bound fraction from normal B cells contained very little eIF4G and eIF4A. However, all the MCL cell lysates were enriched with eIF4G, eIF4E and eIF4A (Figure 1A). In order to determine the association of eIF4E with eIF4G, we repeated this experiment by pulling down eIF4G from the cell lysates of MCL cell lines and demonstrated that the immunoprecipitates of eIF4G fraction in MCL cell lysates were indeed enriched compared to normal B cells and IgG control (Supplemental Figure 1A). A pull down assay using eIF4E antibody demonstrated that immunoprecipitates of the eIF4E fraction were enriched in malignant B cells, suggesting reciprocal binding between eIF4G and eIF4E in MCL cells (Supplemental Figure 1B). Overall, these data demonstrate that the cap bound fraction from lymphoma cells was enriched with eIF4G, eIF4E and eIF4A, demonstrating that aggressive lymphoma B cells exist in a translationally activated state.

### Effect of eIF4E depletion or overexpression on the eIF4F complex, cell growth and global protein translation

To further explore if the alterations in eIF4E availability determine the integrity of the eIF4F complex, we depleted eIF4E through shRNA and performed a cap affinity assay to assess the effect on the eIF4F complex. HEK293^eIF4E/shRNA^ and HEK293^con shRNA^ stable cells were immunoprecipitated with m7GTP beads and immunoblotted with eIF4G, 4E-BP1 and eIF4E antibodies. eIF4E inhibition reduced the association of eIF4G and 4E-BP1 without affecting whole cell lysate (10% input) (Figure 1B).

On the other hand, ectopic expression of eIF4E increased the binding of eIF4G and 4E-BP1 to cap in wild type (HEK293^eIF4E/WT^), but not in cap mutant (HEK293^cap mutant^) cells (Figure 1C). Surprisingly, wild type-eIF4E (eIF4E ^WT^) was not able to increase the global protein translation (Data not shown). Colony formation in HEK293^eIF4E/shRNA^ was significantly reduced and a 70% reduction was observed in the eIF4E-depleted cells (Figure 1D).

### Mechanism of overactivation of translation initiation complex eIF4F complex

Formation of the cap dependent translation complex is dependent upon several factors such as availability of eIF4E due to PI3K/mTOR pathway activation, hyperphosphorylation of 4E-BP1, and eIF4E hyperphosphorylation. We sought to determine which factor(s) is responsible for the activated eIF4F complex in aggressive lymphoma.

**Figure 2:**
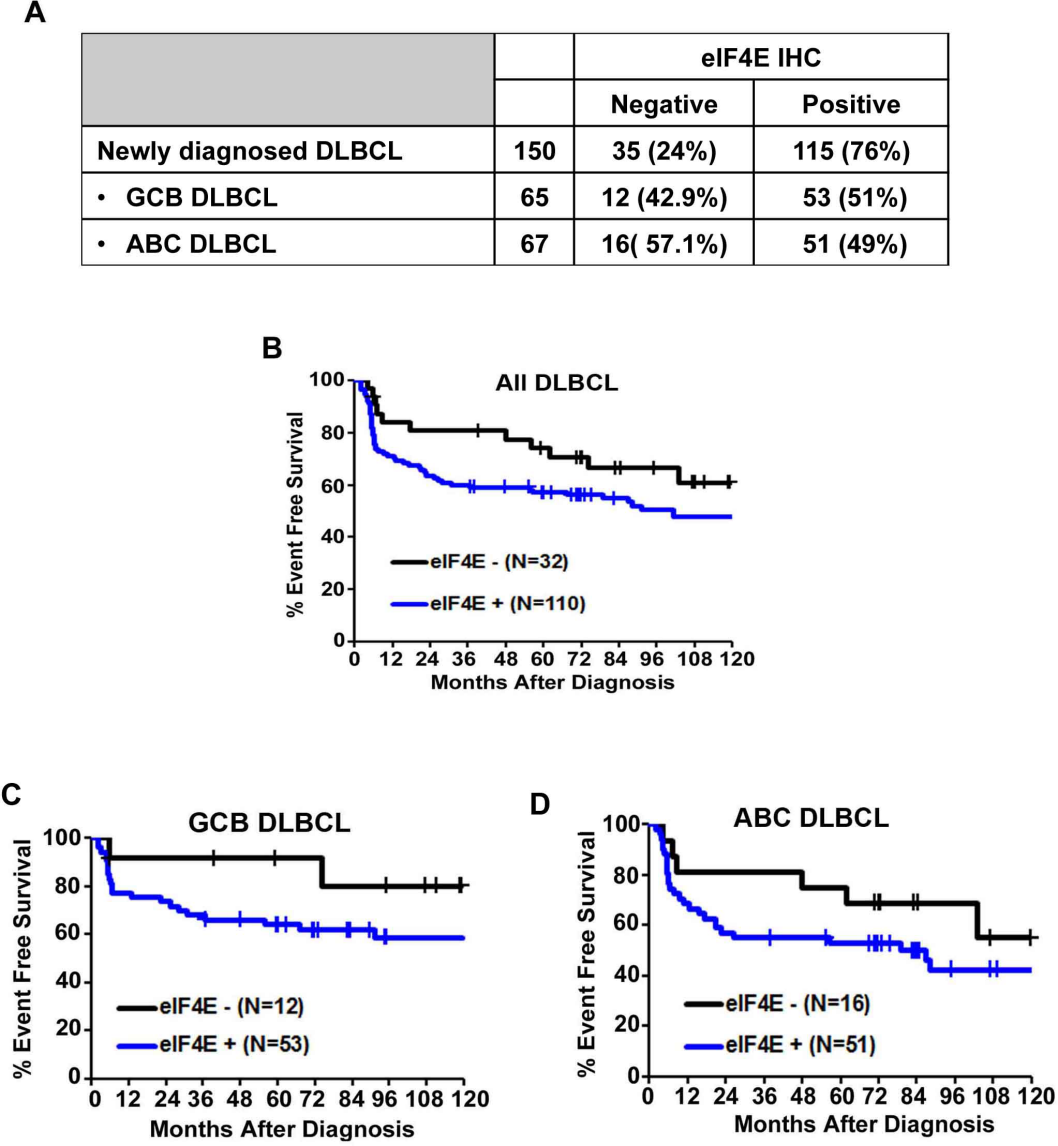
Expression of eIF4E in DLBCL subtypes **(A)** Results of eIF4E IHC staining in all DLBCL and GCB and ABC DLBCL subtypes. **(B)** A Kaplan-Meier (KM) curve for EFS in patients with DLBCL based on the expression of eIF4E is shown. **(C–D)** KM survival curves for GCB (C) and ABC (D) DLBCL subtypes.

We first examined whether there was evidence for eIF4E expression in untreated DLBCL tumor samples using a DLBCL TMA for IHC. The expression of eIF4E was assessed semi-quantitatively as follows; negative (< 10% of cells eIF4E positive) or positive (>10% tumor cells eIF4E positive). Overall, 77% (110/142) patient samples were eIF4E positive (Figure 2A). The eIF4E expression frequencies were similar for germinal center B (GCB) and activated B cell (ABC) tumors (Figure 2A). eIF4E overexpression was significantly associated with stages III and IV of Ann Arbor staging system (Table 1) and with an inferior EFS (*p*-value = 0.12) (Figure 2B). eIF4E expression predicted inferior EFS both in patients with GCB (*p*-value = 0.16) (Figure 2C) or ABC (*p*-value = 0.22) tumor subtypes (Figure 2D).

**Table 1:** Characteristics of the 142 patients with untreated diffuse large B-cell lymphoma used for eIF4E immunohistochemistry

	eIF4E IHC			
	Negative (*N* = 32)	Positive (*N* = 110)	Total (*N* = 142)	*P* value
**Age Group**				0.2282
< = 60 >60	19 (59.4%) 13 (40.6%)	52 (47.3%) 58 (52.7%)	71 (50.0%) 71 (50.0%)	
**Gender**				**0.0197**
Female Male	8 (25.0%) 24 (75.0%)	53 (48.2%) 57 (51.8%)	61 (43.0%) 81 (57.0%)	
**Histology**				0.3491
DLBCL Mediastinal large B-cell	31 (96.9%) 1 (3.1%)	109 (99.1%) 1 (0.9%)	140 (98.6%) 2 (1.4%)	
**Ann Arbor Stage Group**				**0.0117**
I-II III-IV	19 (59.4%) 13 (40.6%)	38 (34.5%) 72 (65.5%)	57 (40.1%) 89 (59.9%)	
**LDH**				0.3303
Missing < = Normal >Normal	3 16 (55.2%) 13 (44.8%)	12 44 (44.9%) 54 (55.1%)	15 60 (47.2%) 67 (52.8%)	
**Number of extranodal disease sites**				0.5629
< = 1 >1	26 (81.3%) 6 (18.8%)	94 (85.5%) 16 (14.5%)	120 (84.5%) 22 (15.5%)	
**ECOG Performance Status**				0.2878
<2 > = 2	27 (84.4%) 5 (15.6%)	83 (75.5%) 27 (24.5%)	110 (77.5%) 32 (22.5%)	
**International Prognostic Index**				0.2588
Low risk (0–1 point) Low intermediate risk (2 points) High intermediate risk (3 points) High risk (4–5 points)	15 (46.9%) 10 (31.3%) 6 (18.8%) 1 (3.1%)	35 (31.8%) 35 (31.8%) 26 (23.6%) 14 (12.7%)	50 (35.2%) 45 (31.7%) 32 (22.5%) 15 (10.6%)	
**Bulky disease**				0.7220
Yes No Unconfirmed	5 (15.6%) 27 (84.4%) 0 (0.0%)	15 (13.6%) 93 (84.5%) 2 (1.8%)	20 (14.1%) 120 (84.5%) 2 (1.4%)	
**B symptoms**				0.1484
No Yes	20 (62.5%) 12 (37.5%)	83 (75.5%) 27 (24.5%)	103 (72.5%) 39 (27.5%)	

LDH, lactate dehydrogenase

Phosphorylated 4E-BP1 dissociates from eIF4E and contributes to the deregulation of eIF4F complex. Next, we assessed the constitutive phosphorylation status of p4E-BP1^Thr37/46^, p4E-BP1^Thr70^ and p4E-BP1^ser65^ in normal B cells compared to MCL (Jeko, Mino, Granta and JVM2) cell lines. All four MCL cell lines over-expressed hyper-phosphorylated 4E-BP1 at all the 3 sites; however, normal B cells have hypo-phosphorylated 4E-BP1 (Supplemental Figure 2). Overall, these data suggest that high levels of eIF4E along with hyper-phosphorylated 4E-BP1 exist in the lymphoma cells and contribute in the formation of active eIF4F complex.

### Active site dual mTORC1/mTORC2 inhibitor CC214-1 blocks the eIF4F complex and cap dependent protein translation

**Figure 3:**
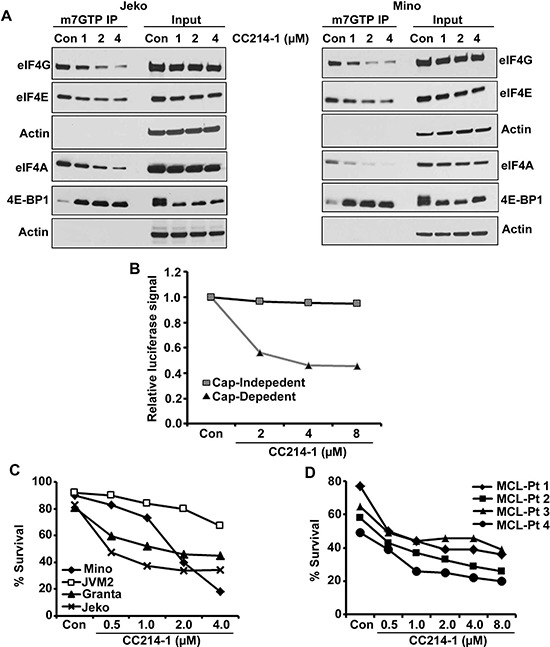
Effect of the dual mTOR kinase inhibitor CC214-1 on eIF4F complex **(A)** Effect of CC214-1 on eIF4F complex integrity by *in vitro* cap affinity assay in Jeko and Mino cells. **(B)** Cap dependent and independent translation was assessed by luciferase reporter assay by the use of bicistronic plasmid in the presence of CC214-1. Bars represent mean ± SD from 3 replicates. **(C)** Survival was evaluated in Mino, JVM2, Granta and Jeko cells treated with CC214-1. **(D)** Effect of CC214-1 on survival inhibition in malignant cells from fresh MCL patient was evaluated using annexin V/PI staining and flow cytometry.

Since there is currently no drug that can directly inhibit the deregulated eIF4F complex in cancer cells, we assessed the effect of the next generation dual mTOR inhibitor CC214-1 on the translation initiation complex downstream of mTOR. We performed a cap affinity assay in Jeko and Mino MCL cells treated with CC214-1. CC214-1 caused a dose dependent inhibition in the association of eIF4G and eIF4A with eIF4E in both Jeko and Mino cells without much effect on the global quantity of these proteins (Figure 3A). Further evidence that CC214-1 was inhibiting the eIF4F complex formation was the increase in eIF4E bound 4E-BP1 after CC214-1 treatment of Jeko and Mino cells. Total levels of 4E-BP1 in the 10% input of CC214-1 treated immunoprecipitates were decreased (Figure 3A). Rapamycin, an mTORC1 inhibitor failed to alter the binding of eIF4G and 4E-BP1 to cap in Mino cells (Supplemental Figure 3A). We, then, evaluated the consequences of CC214-1 treatment on the 4E-BP1 phosphorylation in MCL cell lines. We observed a dose dependent decrease in the hyper-phosphorylated form of 4E-BP1 in response to CC214-1 but not with mTORC1 inhibitor at the given concentration. This effect was observed in multiple 4E-BP1 phosphorylation sites including 4E-BP1 at serine 65 in Jeko cells (Supplemental Figure 3B). However, both CC214-1 and mTORC1 inhibitor inhibited the S6 ribosomal protein phosphorylation. CC214-1 inhibited global protein translation in both Jeko and Mino cells (Supplemental Figure 4).

Effect of CC214-1 on the cap-dependent translation was examined using bicistronic plasmids. In this plasmid, renilla luciferase gene represents cap-dependent protein translation, and firefly luciferase gene control cap-independent translation (pRF). CC214-1 inhibited the cap-dependent translation in a dose dependent manner with limited effect on the cap-independent translation as shown by luciferase assay (Figure 3B).

The effect of CC214-1 on lymphoma cell survival was first evaluated in MCL cell lines such as Mino, Jeko, JVM2 and Granta. CC214-1 treatment produced a dose dependent inhibition of survival in all the 4 MCL cell lines tested (Figure 3C). In order to overcome any differences regarding microenvironment and growth rate between MCL cell lines and patient samples, we treated malignant cells from 4 different MCL patients with CC214-1 *in-vitro*. Similar to the cell lines, CC214-1 treatment caused inhibition of patient tumor cell survival (Figure 3D).

### Effect of CC214-1 on translation of eIF4E targets

**Figure 4:**
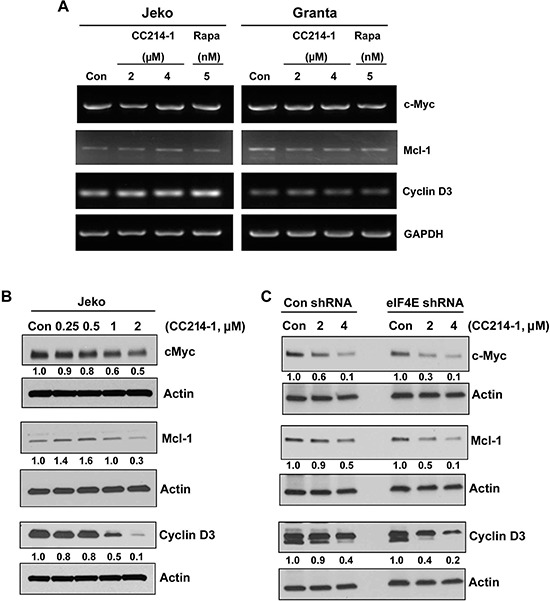
Effect of mTORC1/mTORC2 inhibitor CC214-1 on expression of eIF4E sensitive mRNAs **(A)** RT–PCR was performed in the CC214-1 treated Jeko and Granta cells using specific primers for cyclin D3, Mcl-1 and c-Myc. GADPH is shown as a loading control. The experiment was repeated 3 times. **(B)** Cyclin D3, Mcl-1 and c-Myc protein expression was assessed by western blotting in Jeko cells after treatment with various concentrations of CC214-1. **(C)** Cyclin D3, Mcl-1 and c-Myc protein expression was assessed in eIF4E shRNA stably transfected HEK293 cells in the presence and absence of CC214-1.

Long, highly structured 5′-UTRs are typical of proto-oncogene mRNAs such as c-Myc, Mcl-1 and Cyclin D3 and considered eIF4E sensitive.[3, 14] To evaluate the effects of treatment with CC214-1 on the transcription of eIF4E known targets, we performed qualitative RT-PCR using specific primers for cyclin D3, Mcl-1 and c-Myc. CC214-1 did not inhibit the transcription of cyclin D3, Mcl-1 and c-Myc mRNA in Jeko and Granta cells (Figure 4A). However, CC214-1 was able to suppress c-Myc and cyclin D3 protein level in a dose dependent manner. Suppression of Mcl-1 was detectable only after treatment with higher concentrations of CC214-1 (Figure 4B).

Next, we assessed the effect of the inhibition of eIF4E on the response to CC214-1 on c-Myc, Mcl-1 and cyclin D3 protein level. Our data demonstrate that eIF4E inhibition increased the sensitivity of CC214-1 to inhibit c-Myc, Mcl-1 and cyclin D3 protein levels at 2 μM concentration. (Figure 4C). Moreover, 4 μM CC214-1 still affected sensitivity of Mcl-1 and Cyclin D3 to eIF4E inhibition; however, we have not observed the same response in c-Myc protein (Figure 4C).

### CC214-1 reduces c-Myc, Mcl-1 and cyclin D3 translation by inhibiting polysomal RNA

**Figure 5:**
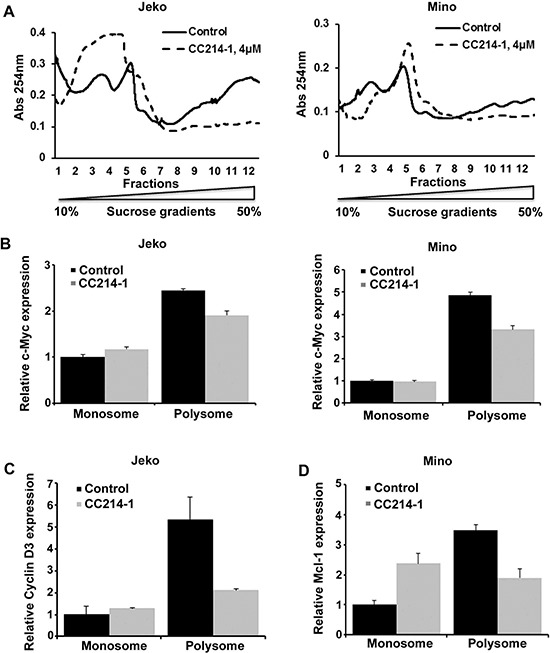
Effect of CC214-1 on polysomal RNA **(A)** Polysomal analysis was performed as described in method section in CC214-1 (4 μM) treated Jeko and Mino cells. Monosomal and polysomal fractions were pooled and Q-PCR was performed for c-Myc (**B**) cyclin D3 (**C**) and Mcl-1 (**D**). Bars represent mean ± SD from 3 replicates. The Q-PCR data were normalized to GADPH.

Polysomes are groups of actively translating ribosomes that are held together by a single mRNA transcript. Polysome profiling was carried out in Jeko and Mino cells as described in the Methods section. We investigated the mechanism of the translation inhibition function by CC214-1 using sucrose density gradient centrifugation. CC214-1 caused a decrease in the RNA absorbance at the heavier gradients suggesting polysome inhibition (Figure 5A) in Jeko and Mino cells.

Polysomal mRNA levels of c-Myc, Mcl-1 and cyclin D3 were assessed in response to CC214-1 in the pooled monosomal and polysomal fractions. Indeed, in cells treated with CC214-1 there was a reduction of c-Myc (Figure 5B), cyclin D3 (Figure 5C) and Mcl-1 (Figure 5D) mRNAs only in the polysomal fractions in MCL cells. These data suggest that the dual mTOR inhibitor CC214-1 interferes with translation process by inhibiting the function of actively translating ribosomes.

### Effect of increased or decreased eIF4E availability on CC214-1 mediated effect on eIF4F complex

**Figure 6:**
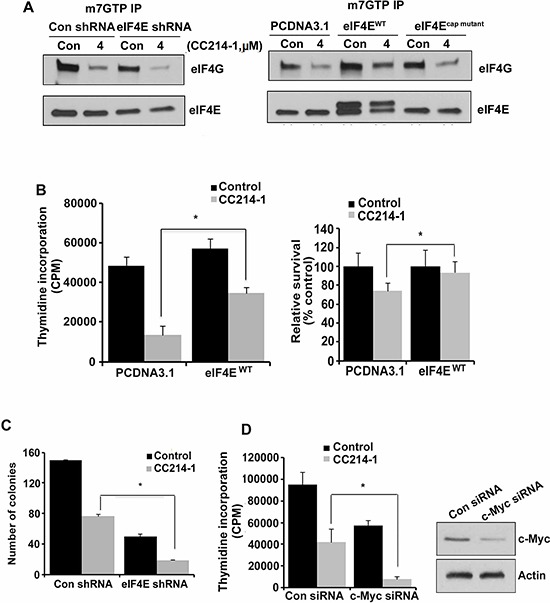
Effect of CC214-1 treatment on eIF4F complex and cell growth in the presence of decreased or increased eIF4E **(A, left panel)** *In vitro* cap-affinity assay (mGTP) in HEK293 cells stably transfected by eIF4E shRNA and control shRNA plasmids. **(A, right panel)** *In vitro* cap-affinity assay was performed in the HEK293 cells transiently transfected by PCDNA3.1 eIF4Ecap mutant and eIF4E^WT^. The experiments were repeated 3 times. **(B)** HEK293 cells transiently transfected by PCDNA3.1 eIF4E^cap mutant^ and eIF4E^WT^ were treated with CC214-1 (1 μM) and thymidine incorporation assay (left panel) and flow cytometry (right panel) were performed. Bars represent mean ± SD from 3 different experiments. **(C)** Colony forming assay in CC214-1 (1 μM) treated HEK293 cells, stably transfected with eIF4E shRNA and control shRNA. Bars represent mean ± SD from 3 replicates. **(D)** HEK293 cells after transfection by control and c-Myc siRNAs, were treated with 1 μM CC214-1 and thymidine incorporation was performed. Bars represent mean ± SD from 3 replicates. The experiment was repeated 3 times. The effect of the siRNAs on c-Myc expression was analyzed by western blot.

eIF4E levels have been found to be elevated in several types of cancer.[15–18] To elucidate the role of eIF4E availability in the response to CC214-1 on eIF4F complex, eIF4E was knocked down and then the cells were treated with CC214-1. CC214-1 was able to inhibit the binding of eIF4E and eIF4G to cap in both eIF4E shRNA as well as in the control shRNA cells; however, inhibition was slightly more in the eIF4E depleted cells (Figure 6A, left panel). Next, we sought to determine the CC214-1 effect on eIF4F complex integrity while eIF4E is overexpressed. Wild type eIF4E was transiently expressed and assays for cap affinity, proliferation, and apoptosis were performed. CC214-1 suppressed the binding of eIF4G to cap in HEK293^cap mutant^ and HEK293^PCDNA3.1^ cells. CC214-1 was able to inhibit binding of eIF4G to cap in cells over-expressing wild type-eIF4E but the binding was less inhibited as compared to HEK293^PCDNA3.1^ and HEK293^cap mutant^ (Figure 6A, right panel).

Furthermore, the assay revealed a 72% inhibition in the cell proliferation in the plasmid alone cells as compared to untreated cells. However only 39% inhibition was observed in the proliferation in the eIF4E overexpressing cells as compared to untreated cells (Figure 6B, panel left). To further confirm the effect of high eIF4E availability on cell survival after treatment with CC214-1, flow cytometry was performed. HEK293^PCDNA3.1^ cells showed 26% reduction of survival in plasmid alone cells as compared to the untreated control, however eIF4E overexpressing cells showed only 7% inhibition in survival as compared to the untreated control (Figure 6B, panel right).

In eIF4E-shRNA cells, treatment with CC214-1 was able to significantly inhibit colony formation (70%) as compared to control shRNA cells (50%) (Figure 6C). To evaluate the effect of c-Myc deregulation in dual mTOR inhibition, we treated cells with either c-Myc or control siRNA for 48 hours and proliferation was performed. Silencing the expression of c-Myc resulted in decreased proliferation. Moreover treatment with CC214-1 inhibited cell proliferation 56% in plasmid alone cells as compared to the untreated cells, whereas 83% suppression of proliferation was observed in HEK293^c-Myc siRNA^ cells as compared to the untreated cells (Figure 6D). Western blotting shows the successful knockdown of c-Myc protein (Figure 6D, right).

## DISCUSSION

The eIF4F complex, an important downstream target of the mTOR pathway, plays a critical role in the regulation of cap-dependent translation. Herein we show that the active translation initiation eIF4F complex (eIF4E-eIF4G-eIF4A) bound to cap is deregulated in aggressive lymphoma cells whereas expression of active eIF4F assembly in normal B cells is low. In the present study we demonstrated that 76% of tumors from newly diagnosed DLBCL patients (*n* = 150) express eIF4E by IHC and that this expression predicts shorter EFS. These results are consistent with other studies in solid tumors that demonstrated inferior patient outcome when the tumor cells overexpressed eIF4E.[19] The positive expression of 4E-BP1[20] and eIF4E[17] in MCL and DLBCL tumors by IHC has previously been reported but without relationship to patient outcome. We have previously demonstrated in a separate cohort of relapsed MCL patients that the 30% (9/30) of patients with p4E-BP1+ tumors had a shorter progression-free survival when treated with the mTORC1 inhibitor temsirolimus and rituximab.[21]

In order to address the question whether eIF4E availability is necessary for the formation of m7GTP-eIF4E-eIF4G complex we performed cap-affinity assays in transfected cells. Based on our results, the level of eIF4E is critical for the formation of the translation initiation assembly as its availability is correlated to the formation of active eIF4F assembly in a linear form. Consistent with previous studies, the eIF4E level did not affect global protein translation[22–24], whereas knocking down eIF4E decreased the ability of the cells to form colonies.[25, 26] Yanagiya et al[24] have attributed the observation that global protein translation is not decreased despite the strong eIF4E reduction to the fact that hypo-phosphorylated 4E-BP1 levels are also reduced after knocking down eIF4E.

Since a direct inhibitor targeting the eIF4E level is currently not available, several studies have aimed at targeting eIF4E using indirect approaches.[27, 28] An important upstream regulator of the eIF4E pathway is mTOR, a serine/threonine protein kinase that functions by phosphorylating eIF4E binding proteins (4E-BPs).[29] The mTOR pathway has been shown to be constitutively activated in a high percentage of B-cell lymphomas, [11, 30, 31] however, some lymphomas develop resistance to mTORC1 inhibition. Indeed, multiple clinical trials report modest tumor response rates to single-agent mTORC1 inhibitors in many types of relapsed NHL, Hodgkin lymphoma and Waldenstrom macroglobulinemia.[9] Gupta et al have demonstrated before that mTORC1 inhibition with rapamycin resulted only in modest antiproliferative effect in aggressive lymphoma cells. In the same study, this was attributed to resistance due to high expression of mTORC2 and subsequent Akt and eIF4E phosphorylation.[11] Furthermore, Gupta et al have recently provided the first demonstration of antiproliferative and proapoptotic properties of a dual mTORC1/mTORC2 inhibitor in lymphoma through Akt pathway inhibition.[13] In the studies reported herein, we sought to understand the effect of CC214-1, a next-generation mTORC1/mTORC2 inhibitor on translation pathway associated with eIF4E. We have also investigated the benefits of a potential use of the drug in patients. Dual mTOR inhibition targets cap-dependent translation by decreasing the association between eIF4F assembly and cap, induces apoptosis, and inhibits protein expression of c-Myc and cyclin D3 without much effect on their mRNA expression.

Since transcription was unaffected, a polysome analysis in lymphoma cell lines was carried out in order to explore the mechanism through which this dual mTOR inhibitor suppresses translation. Our data show that CC214-1 treatment causes suppression of mRNA polysome levels without much effect in monosomal mRNA. In addition, CC214-1 inhibits c-Myc, Mcl-1, and cyclin D3 translation through suppression of the highly translationally active polysomes. These data indicate that CC214-1 acts through inhibition of translation initiation whereas joining of the ribosomes to the pre-initiation complex is unaffected. Of note, we have also observed that in some occasions treatment with high concentration of drug might decrease total mRNA in monosomes. Although our results indicate that CC214-1 acts mainly by inhibiting cap-dependent translation, the latter finding could be attributed to several factors, as post-treatment cell viability was more than 90% in all cases. Wall et al[32] have recently reported decreased, rapamycin induced, translation of c-Myc in promyelocytes through inhibition of translation initiation. Decreased translational efficiency of Mcl-1 mRNA after rapamycin treatment has also been shown before.[33]

In summary, this study presents information regarding alterations in the translation control providing, in parallel, a mechanistic basis of targeting cap-dependent translation through next generation mTORC1/mTORC2 inhibitors. The presence of eIF4E expression by IHC should be further evaluated in trials of dual mTOR inhibitors to learn whether the expression predicts response to these agents in lymphoma patients.

## METHODS

**Cell lines.** Mantle cell lymphoma cell (MCL) lines Jeko, Mino, Granta and JVM2 were purchased from ATCC and grown in Roswell Park Memorial Institute medium with 10% fetal bovine serum (FBS). HEK293T cells were grown in DMEM medium with 10% FBS.

**Antibodies and reagents.** CC214-1, a dual mTORC1/mTORC2 inhibitor, was provided by Celgene Pharmaceuticals.[34] Phospho-4EB-P1^Th37/46^, phospho-4E-BP1^Th70^, phospho-4E-BP1^Ser65^, antibodies were purchased from the Cell Signaling Technologies (Beverly, MA, USA). Antibodies for eIF4E, eIF4G, 4E-BP1, cyclin D3, c-Myc and Mcl-1 were also purchased from Cell Signaling Technologies; antibody to Actin was purchased from Santa Cruz (Dalas, TX, USA). 7-Methyl guanosine triphosphate-Sepharose 4B (m7GTP) beads were purchased from GE Healthcare (Buckinghamshire, HP7 9NA UK).

**Patient samples.** Tissue microarrays (TMA) were constructed using triplicate 0.6-mm cores from paraffin-embedded DLBCL tissue blocks (*n* = 142) and included 10 nonmalignant tonsil controls. The TMA was obtained through the University of Iowa/Mayo Lymphoma SPORE. All patients provided written consent for use of their samples and outcome information and the master protocol was approved by the Institutional Review Boards of the Mayo Clinic and University of Iowa. Normal B cells were isolated using CD19 microbeads (Miltenyi Biotec) from peripheral blood mononuclear cells from healthy donors.

**Cap affinity assay (m7GTP).** Briefly eight to ten million cells were washed with ice-cold 1x PBS (Phosphate-Buffered Saline) followed by lysis with cap binding buffer (20 mM pH7.2 Hepes, 1mM EDTA, 100 mM KCL, 10% v/v glycerol, 7 mM 2-mercaptoethanol, 50 mM glycerophosphate, 50 mM Sodium fluoride) using four freeze-thaw cycles. 7-Methyl GTP-Sepharose beads were added to the lysates and incubated at 4^°^C for 3 hours with rotation. Subsequently, the beads were washed to dissolve the protein bound to the beads and western blot was performed with specific antibodies.

**RNA extraction, cDNA synthesis and RT-PCR.** RNA extraction, cDNA synthesis, qualitative and quantitative RT-PCRs (Q-PCR) were performed as previously described.[13] The following primers were used for Q-PCR: c-Myc forward, 5′- GACGACGAGACCTTCATCAAAAAC-3′, and c-Myc reverse, 5′-AGGCCAGCTTCTCTGAGAC-3′; Mcl-1 forward, 5′- CTGGGATTGAGAGGTTGATGAATG-3′, and Mcl-1 reverse, 5′- TGCCCAATCAGAGCCCATTATTTG-3′; Cyclin D3 forward, 5′-GGCCCTCTGTGCTACAGA TTATACC-3′; Cyclin D3 reverse, 5′-CGCAGGCAGTC CACTTCAGTG-3′; Glyceraldehyde-3 phosphate dehydrogenase (GADPH) forward, 5′-ATCACCATCTTCC AGGAGCG-3′ and GADPH reverse, 5′-CAAATGAGC CCCAGCCTTC-3′.

**Transient transfection for adherent cells.** For plasmid transfection, cells were transfected with 5 μg of plasmids such as PCDNA3.1, eIF4E^WT^ and eIF4E^cap mutant^ using lipofectamine 2000 reagent (Invitrogen, Grand Island, NY, USA) and Opti-MEM medium. For small interfering RNA (siRNA) experiments, cells were transfected with 50 nM c-Myc siRNA and control siRNA using lipofectamine 2000 reagent.

**Western blotting.** Western blotting was performed as described before.[35] Densitometry was performed using AlphaImager Software (Alpha Innotech, San Leandro, CA).

**Colony formation assay.** HEK293T cells were treated accordingly and were incubated for 6 days. Cells were washed, fixed and stained with a staining solution (0.06% coomassie blue, 45% methanol and 10% acetic acid). Colonies were counted using colony count program on Alphaimager IS-3400 (Alpha Innotech, CA, USA).

**^3^H leucine incorporation assay.** 1 × 10^5^ cells were treated accordingly for 48 hours. Then cells were incubated for 24 h in the presence of 2 μCi/ml leucine. 10% trichloroacetic acid was added and cells were incubated for another 30 min at 4ºC. Cells were lysed with 0.4N Sodium hydroxide and transferred in triplicates in a 96 well plate. ^3^H leucine radioactivity was determined by liquid scintillation spectrometer.

**Cell survival by annexin V/Propidium iodide.** Annexin V/PI binding was assayed as described.[11]

**Luciferase cap dependent transfection.** Jeko cells were transfected with luciferase plasmid (pRF) using Amaxa Cell Line Nucleofector kit as per manufacturer*'*s instructions. Plasmid pRF was a kind gift from Dr. Gregory Goodall.[36] The luciferase assay was performed using a dual-luciferase report assay kit (Promega, Madison, WI, USA) as per the manufacturer*'*s instruction.

**Thymidine incorporation.** ^3^H-thymidine incorporation assay as described before.[37]

**Polysome analysis.** Polysome analysis was performed as described earlier with some modifications.[38, 39] 70–90 million cells were seeded and treated accordingly overnight. Cells were treated with 0.1mg/ml cycloheximide (CHX) for 3min at 37°C and then, cells were collected. All subsequent procedures were carried out on ice. Cell lysates were loaded on10–50% sucrose density gradients and sedimented for 3hrs at 35000 rpm in a Beckman SW40 rotor at 4ºC. Gradients were fractionated, and the optical density (OD) at 254 nm was counted using density gradient fractionating system (Brandel, MD). RNA was precipitated using lithium chloride.

**Immunohistochemistry (IHC).** DLBCL cases (*n* = 132) were classified into GCB or non-GCB molecular type based on the Hans algorithm applied to paraffin-embedded tumor samples.[40] IHC on tissue microarray was performed using eIF4E antibody and IgG control as previously described.[41] A 10% cut off was chosen for eIF4E positivity. Slides were reviewed by a hematopathologist (A.D).

**Statistics.** Event-free survival (EFS) was defined as time from diagnosis to progression, relapse, re-treatment, or death due to any cause. Associations between eIF4E status and EFS were assessed using Cox proportional hazards models and Kaplan Meier curves. The *p*-value for *in-vitro* data was calculated using the means from 3 different experiments (two-tailed unpaired Student's *t* test).

## SUPPLEMENTARY FIGURES


