# Supplementary material for: Translation initiation complex eIF4F is a therapeutic target for dual mTOR kinase inhibitors in non-Hodgkin lymphoma
Source: Oncotarget. 2015 Mar 20;6(11):9488–501. doi: 10.18632/oncotarget.3378 (PMC4496233; doi:10.18632/oncotarget.3378)
Supplement: Supplementary file 1 [file oncotarget-06-9488-s001.pdf]

SUPPLEMENTARY FIGURES

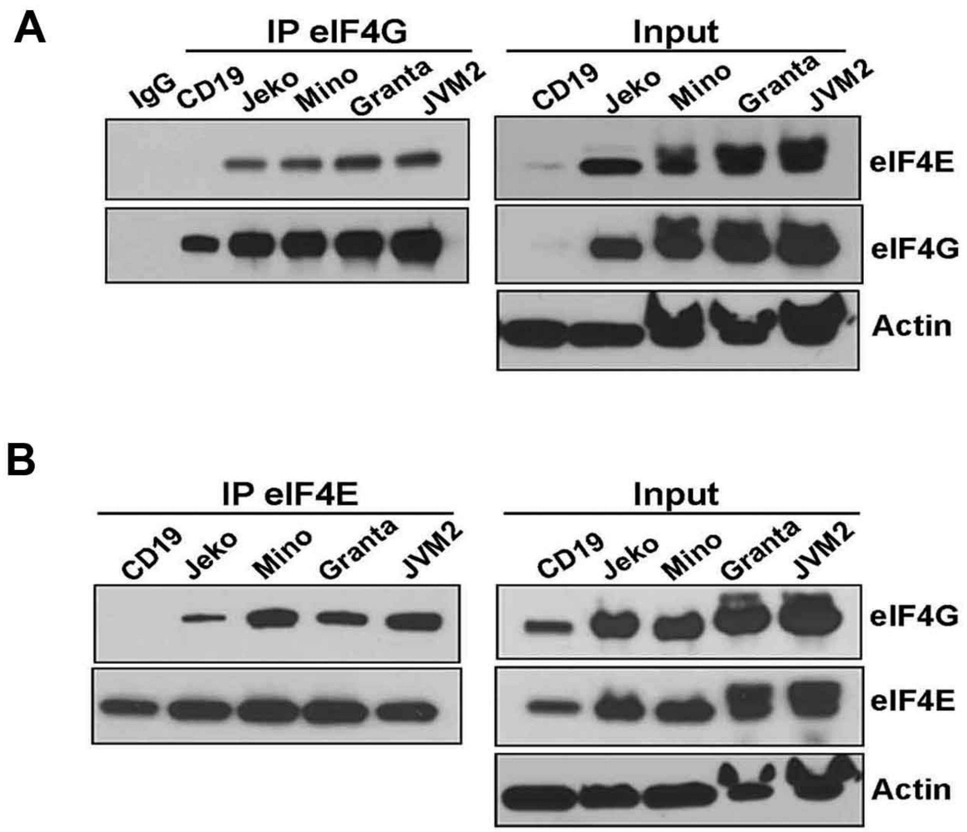

Supplementary Figure 1: (A) Immunoprecipitation (IP) with eIF4G and IgG antibodies was performed in MCL cell lines and normal B cells and western blotting was performed using eIF4G and eIF4E antibodies. (B) IP was performed in MCL cells with eIF4E antibody, followed by immunoblotting with eIF4G and eIF4E antibodies.

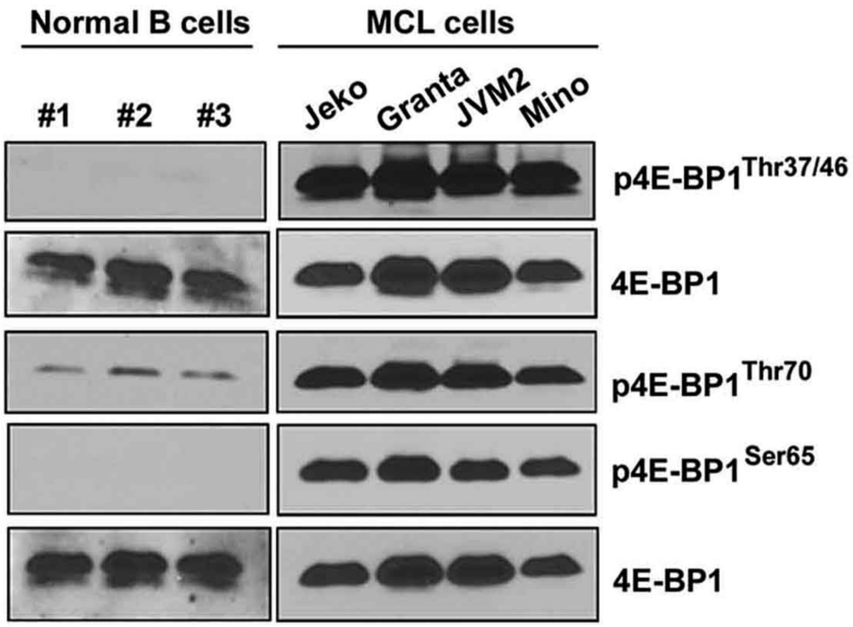

Supplementary Figure 2: Constitutive phosphorylation of 4EBP1 was assessed in the MCL cell lines and in normal B cells by western blotting.

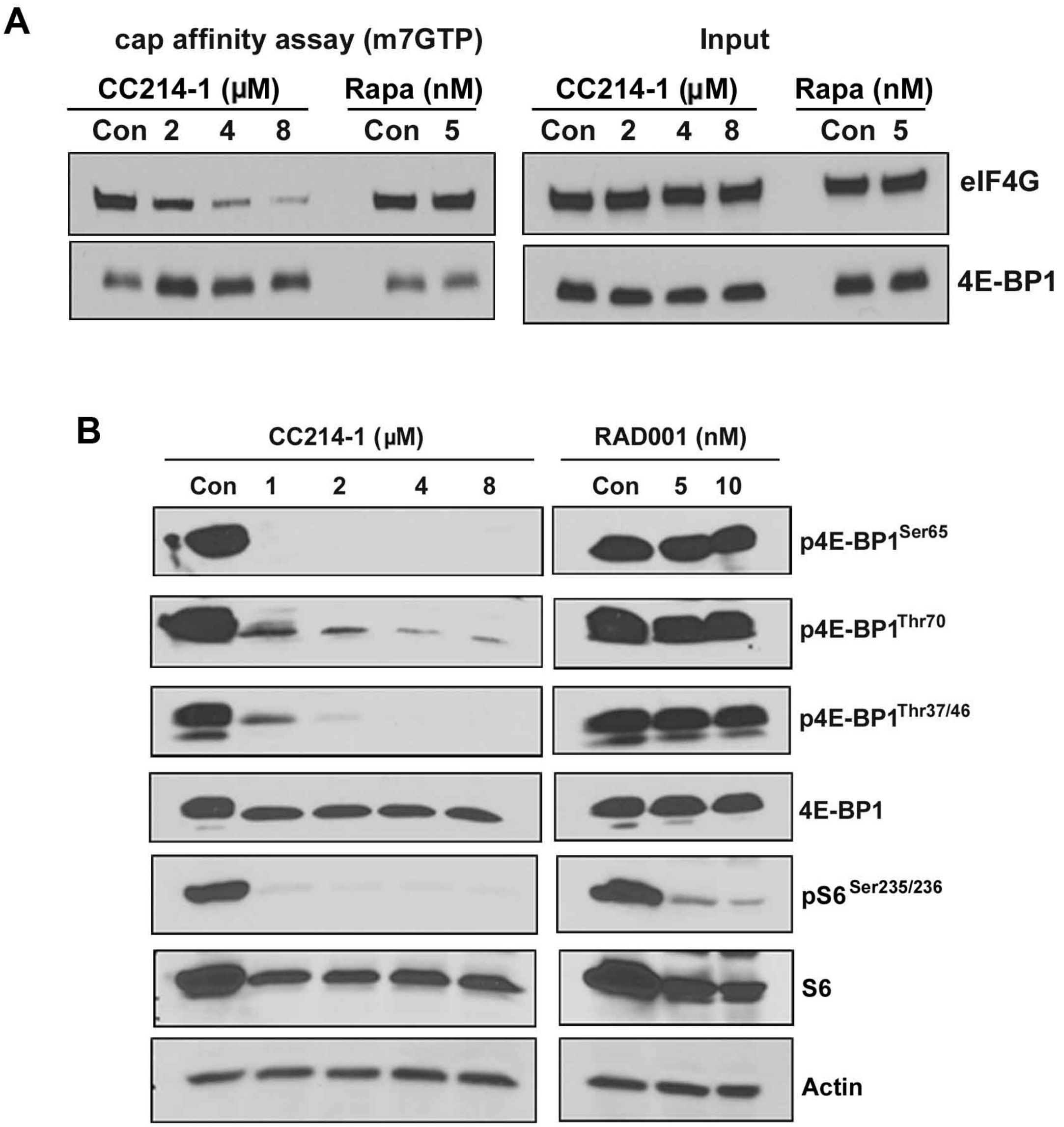

Supplementary Figure 3: (A) In vitro cap-affinity assay as carried out in Mino cells after treatment with CC214-1 or Rapamycin. The experiment was repeated 2 times. (B) Western blot analyses were performed to examine the effect of CC214-1 on phosphorylation of 4EBP1 and S6. Actin is shown as a loading control.

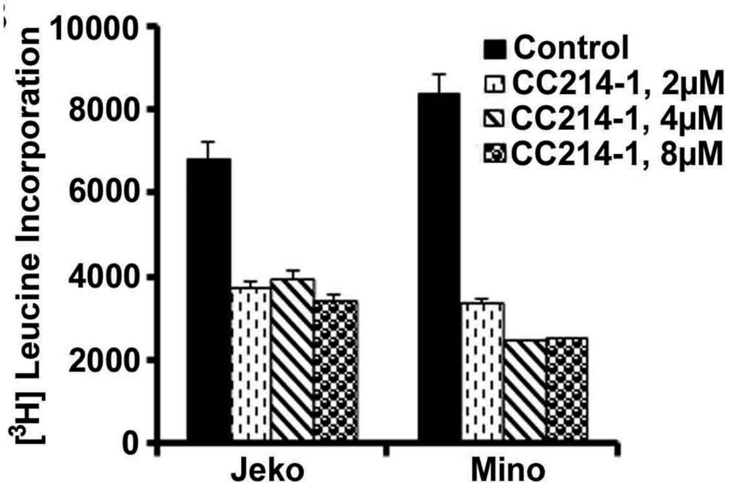

Supplementary Figure 4: ^{3}H **Leucine incorporation assays were performed in the Jeko and Mino cells as the readout for global protein translation. Bars graphs represent mean ± SD from 3 replicates.**
